# Extended Reality in Revolutionizing Neurological Disease: A New Era for Chronic Condition Treatment

**DOI:** 10.7759/cureus.67633

**Published:** 2024-08-23

**Authors:** Hariharan V, Malini Prithiva Kumari PK, Rajanandh MG

**Affiliations:** 1 Department of Pharmacy Practice, SRM College of Pharmacy, SRM Institute of Science and Technology, Chennai, IND

**Keywords:** neuroplasticity, patient-centred care, pharmacy practice, mixed reality, augmented reality, virtual reality

## Abstract

Extended reality (XR), which includes virtual reality (VR), augmented reality (AR), and mixed reality (MR), provides promising advancements in managing chronic neurological disorders such as Parkinson's disease (PD), multiple sclerosis (MS), Alzheimer's disease, and stroke. This review examines the impact of XR technologies on neurological care, highlighting their ability to create immersive, interactive environments that enhance rehabilitation through tailored motor and cognitive exercises. XR supports neuroplasticity by providing engaging, contextually relevant exercises and real-time feedback, offering innovative alternatives to traditional methods. The technical issues, clinical validation, and accessibility must be addressed despite the potential benefits. Future developments should focus on refining XR applications, integrating them with complementary technologies, and establishing robust policies to guide their effective and ethical use. XR is poised to revolutionize neurological rehabilitation, promising improved patient outcomes and transforming medical training.

## Introduction and background

According to the World Health Organization (WHO), neurological disorders have a profound impact on nearly one billion people worldwide. This extensive category encompasses conditions such as seizures, Alzheimer's, stroke, migraines, brain injury, multiple sclerosis (MS), and Parkinson's disease (PD). Approximately 50 million individuals are affected by epilepsy, while an additional 24 million are affected by Alzheimer's and other dementias. These disorders do not discriminate and influence people across all nations, irrespective of their age, gender, education, or socioeconomic status, contributing to an estimated 6.8 million fatalities annually [1]. These problems are caused by a variety of factors, including head injuries, strokes, and neurodegenerative diseases, including PD and MS [2]. As the global population ages, the prevalence of neurological disorders increases. However, advancements in medical research are offering new hope for those affected by conditions such as Alzheimer's, PD, and MS [3].

Treating neurological disorders often requires complex, long-term drug therapies to alleviate symptoms and slow disease progression. While traditional treatments, such as medications, gene therapies, and stem cell research, have shown promise, they can be tedious, leading to a loss of motivation and a decline in patient outlook on rehabilitation [2]. New technologies, including assistive devices, communication aids, and home automation systems, are being developed to help those with neurological problems live more independently. However, there is no one-size-fits-all approach to treating neurological conditions, as each patient's situation is unique [3].

**Figure 1:**
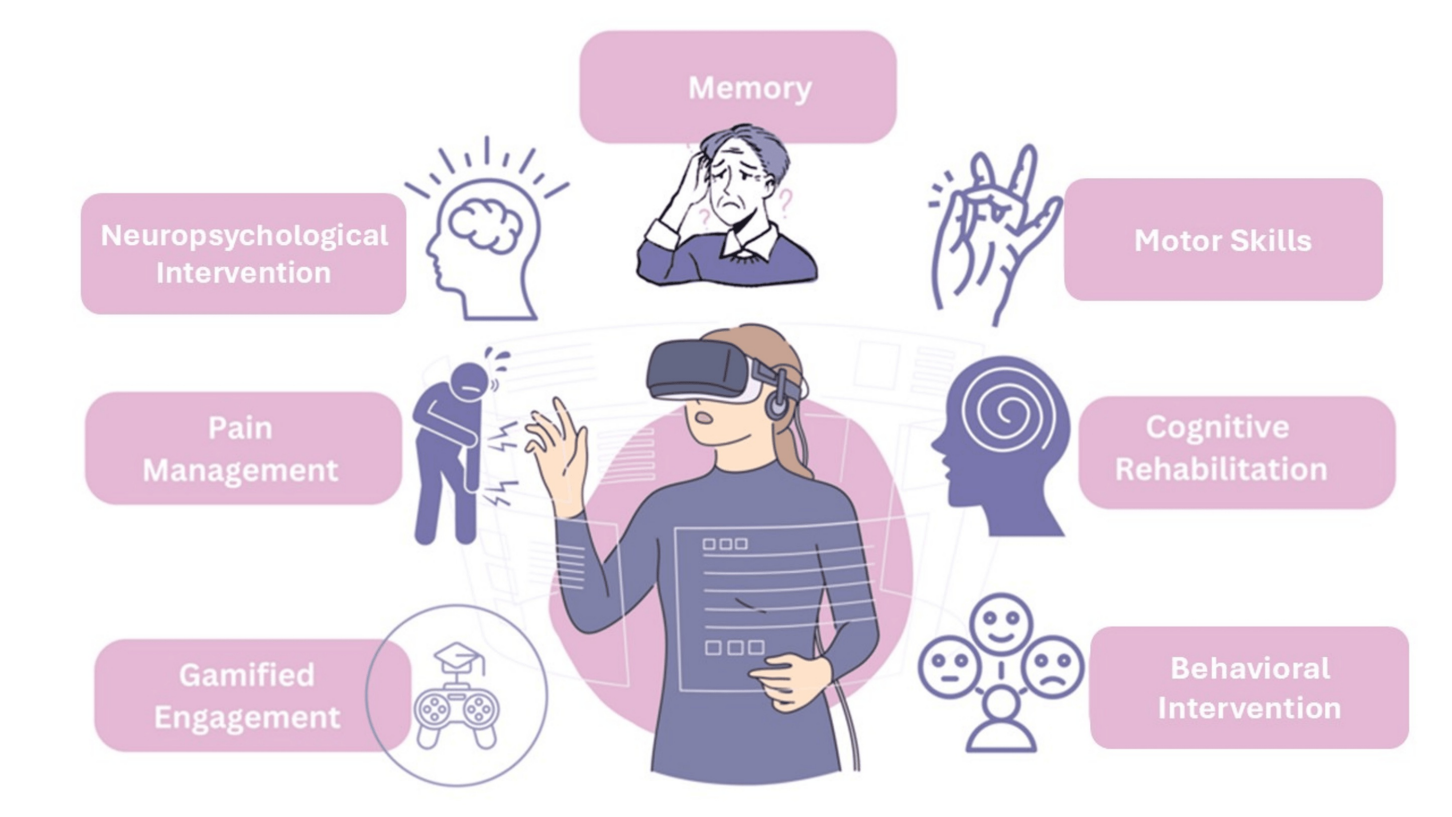
Role of virtual reality in neurological care Image credit: author

In recent years, extended reality (XR) technologies, encompassing virtual reality (VR), augmented reality (AR), and mixed reality (MR), have emerged as promising tools in neurological care. XR technologies differ from traditional therapies by offering immersive, sensory-rich environments that provide real-time feedback, enhancing therapeutic engagement and outcomes [4,5]. For example, VR is increasingly recognized for its role in cognitive research, evaluation, and rehabilitation. It allows patients to engage in realistic activities and receive precise performance measurements in a safe, controlled setting (Figure 1). Given the limitations of traditional treatments and the unique benefits XR technologies offer, this narrative review explores its potential, current applications, limitations, and future directions of XR technologies in managing chronic neurological conditions. In examining how XR can complement or enhance traditional therapies, this review aims to shed light on the evolving landscape of neurological rehabilitation and care.

## Review

Overview of extended reality

VR puts users in a completely digital environment, replacing the real world with a simulated one via head-mounted displays (HMDs) such as the PlayStation VR. Users interact with these environments using motion-tracked input devices like controllers and gloves, translating their movements into the virtual space. The experience is powered by advanced software that creates and manages the virtual world, offering applications that range from gaming to complex medical simulations [6,7]. AR improves the real world by superimposing digital information on top of it rather than replacing it, as VR does. This technology is typically accessed through devices such as smartphones, tablets, or AR glasses such as Microsoft HoloLens and Google Glass. AR relies on sensors like cameras and GPS to detect the user's surroundings and position, enabling accurate digital content placement. The experience is driven by software that processes sensor data and renders the digital overlays, with platforms like Apple's ARKit and Google's ARCore leading the way in AR development [8]. MR combines aspects of virtual and AR, allowing real and virtual things to interact in real time. MR provides an enhanced user experience by combining the physical and digital worlds, allowing users to interact with both at the same time.

XR, including VR, AR, and MR, shows great potential in neurology by offering innovative methods for diagnosing, treating, and rehabilitating neurological diseases. These technologies create immersive environments that enhance cognitive assessments and motor rehabilitation, particularly in conditions like stroke. VR environments can replicate real-world tasks and scenarios, providing a controlled setting for cognitive assessments and allowing clinicians to evaluate functions like memory, attention, and executive function with greater ecological validity compared to traditional paper-and-pencil methods [9]. Building on this capability, VR and AR can also create engaging and motivating environments for motor rehabilitation. Patients, particularly those recovering from strokes, can perform repetitive, task-specific exercises within these virtual settings, promoting neuroplasticity and improving motor function, such as upper limb movement [10]. Together, these applications highlight the potential of XR technologies in enhancing various aspects of neurological care.

Applications of XR in neurological disease treatment

Stroke

Stroke is the main cause of global disability, resulting in significant motor and cognitive impairments. In response, VR has emerged as a promising tool for stroke rehabilitation, offering innovative methods for motor recovery and cognitive enhancement. VR-based interventions have shown considerable promise, providing immersive and interactive environments tailored to individual patient needs. These systems offer repetitive, task-specific training, critical for fostering neuroplasticity and functional recovery.

According to a systematic review and meta-analysis by Laver et al. (2017), VR and interactive video gaming can be more successful than traditional therapy in improving upper limb function and activities of daily living (ADL) when used with standard care. Specifically, VR demonstrated a greater impact on upper limb function, with a standardized mean difference of 0.28 [10]. This is further supported by Brunner et al. (2017), which showed that VR-based intervention using the ArmeoSenso system effectively improved motor function in the damaged upper limb of chronic patients with stroke. Together, these findings underscore the growing role of VR in advancing stroke rehabilitation [11]. In addition to motor recovery, VR's role extends to gait and balance training. VR can create safe environments for patients to practice walking and balance exercises. A study by Corbetta et al. (2015), showed that VR training combined with conventional therapy was more effective in improving gait speed, balance, and mobility than conventional therapy alone [12]. VR's impact on motor learning and neuroplasticity is profound. VR provides multisensory feedback, which is crucial for motor learning. Takeuchi and Izumi (2013), reviewed the neurophysiological mechanisms underlying VR-induced neuroplasticity, highlighting how VR can facilitate functional and structural brain changes that support motor recovery [13].

Cognitive impairments are common following a stroke and can significantly impact a patient's functional independence and quality of life. In this context, VR offers unique opportunities for cognitive rehabilitation by providing controlled, engaging environments for targeted cognitive training. VR-based cognitive training programs have shown efficacy in improving attention and executive function. A randomized controlled trial by Faria et al. (2016), demonstrated that a VR-based cognitive rehabilitation program led to significant improvements in attention and executive functions compared to conventional cognitive training [14]. In addition to enhancing attention and executive functions, VR has proven effective in addressing specific cognitive impairments such as unilateral spatial neglect, a common issue following stroke. By creating immersive environments tailored for neglect rehabilitation, VR-based interventions have achieved better outcomes than traditional computer-based training. A study by Kim et al. (2011), found that VR-based spatial neglect therapy resulted in greater improvements in spatial neglect symptoms [15]. Furthermore, VR's capacity to simulate real-world environments offers valuable training for memory and learning skills. Optale et al. (2010), reported that VR-based memory training in elderly patients with memory deficits led to significant improvements in long-term memory and general cognitive functioning [16].

VR's versatility extends to cognitive-motor integration, allowing for simultaneous training of cognitive and motor functions, a crucial aspect of stroke rehabilitation. Maggio et al. (2019) demonstrated that a VR-based cognitive-motor training program improved cognitive and motor outcomes in chronic stroke patients [17]. This integrated approach underscores VR's potential to enhance various facets of cognitive recovery and overall rehabilitation.

Parkinson's Disease

PD is a progressive neurological condition that primarily affects movement, resulting in tremors, stiffness, and difficulties with balance and coordination. AR technology has emerged as a potential tool to assist PD patients in managing their symptoms and improving their daily activities. One of the most promising applications of AR in PD is visual cues for enhancing gait and minimizing freezing of gait (FOG), a prevalent and distressing symptom. AR glasses can project virtual visual cues, such as lines or stepping stones, onto the real-world environment. These cues can help patients initiate and maintain a normal walking pattern. A study by Janssen et al. (2017), found that these AR visual cues significantly reduced FOG episodes and improved gait parameters in PD patients, highlighting AR's potential as an innovative tool in PD management [18]. Beyond gait improvement, AR can provide real-time guidance and assistance for various daily activities that may be challenging for PD patients. AR systems can offer step-by-step instructions for complex tasks, helping patients maintain independence in daily activities. This study demonstrated that at-home training with an AR cueing device improved gait parameters and reduced FOG in PD patients [19].

AR applications can provide interactive environments for motor skill training that is crucial for maintaining and improving motor functions in PD patients. These applications guide patients through exercises that improve motor control, balance, and coordination. This study showed that AR-generated virtual footprints improved walking performance in PD patients, particularly those with more severe gait impairments [20]. While PD primarily affects movement, it can also affect cognitive functions. AR applications can provide cognitive training exercises in real-world contexts. These games can challenge memory, attention, and executive functions while incorporating real-world elements. Although this study focused on older adults in general, it included PD patients and demonstrated that treadmill training augmented with VR reduced fall risk more than treadmill training alone [21]. In addition to physical and cognitive benefits, AR can assist in medication management, a crucial aspect of PD treatment. AR glasses or smartphone apps can provide timely, context-aware reminders for medication intake. While this study used smartphone apps rather than AR specifically, it demonstrates the potential for technology to improve medication adherence in PD patients [22].

Multiple Sclerosis

It is a chronic autoimmune disease that affects the CNS, causing symptoms like muscle weakness, balance difficulty, and coordination impairments. VR technology has shown promise in providing effective balance and coordination training for MS patients. VR environments can simulate various balance exercises and scenarios, providing real-time feedback to patients. These exercises can help improve postural control and reduce the risk of falls. VR-based stability training programs that simulate walking on various surfaces or navigating through challenging obstacles may have a short-term benefit in improving balance and lowering the fear of falling [23]. VR can also provide interactive and engaging exercises that target coordination skills. These exercises can be customized to meet the unique needs and skills of each patient. VR games that require precise hand-eye coordination, such as catching virtual objects or following moving targets [24].

Alzheimer's Disease and Dementia

It is a neurological condition marked by cognitive decline, memory loss, and reduced daily functioning. XR technologies, including VR and AR, offer innovative approaches for cognitive training and memory enhancement. XR can offer deep and engaging cognitive training tasks that target multiple cognitive domains, including memory, attention, and problem-solving. VR environments that simulate daily activities, such as shopping or cooking, to help patients practice and retain cognitive skills [25]. Additionally, AR can provide contextual memory aids and reminders, helping patients with Alzheimer's and dementia navigate their daily lives more independently. AR glasses that display contextual information, such as names of people or locations, to assist with memory recall [26].

Mechanisms of XR in neurological rehabilitation

Neuroplasticity Effects

Neuroplasticity refers to the brain's ability to restructure by generating new neural connections over time. This mechanism is critical for learning, memory, and recovery from brain injury. In this context, VR training has shown significant potential in promoting neuroplasticity, with neuroimaging findings guiding its development. It is a critical mechanism for recovery in neurological rehabilitation, where the repetitive activation of specific neural pathways strengthens synaptic connections and promotes the formation of new neural circuits. It is evident in VR-based hand rehabilitation exercises for stroke patients, where patients repeatedly perform grasping motions in a virtual environment, leading to functional improvements associated with changes in neural activation patterns. Studies have demonstrated that neural activation can shift from contralesional to ipsilesional regions post-training, as seen in the work of Sung Ho Jang et al. (2005) and You et al. (2005) [27,28].

Moreover, XR technologies can provide rich, multimodal sensory experiences that engage multiple brain regions simultaneously with the simultaneous activation of various sensory and motor areas, enhancing neural integration and promoting more comprehensive brain plasticity. It is further supported by systems like an AR platform that combines visual, auditory, and haptic feedback for balance training in multiple sclerosis patients, illustrating how these technologies can contribute to neuroplasticity [29].

Immersive Environments

XR allows for the gamification of rehabilitation exercises, making them more enjoyable and motivating for patients. Game-like elements such as points, levels, and rewards tap into intrinsic motivation systems in the brain, encouraging patients to engage more fully in rehabilitation. This is evident in a VR game where PD patients collect virtual objects by performing specific movements with increasing difficulty as they progress, further enhancing motivation and participation in the therapeutic process [30]. Additionally, XR provides a safe and controlled environment for patients to practice challenging tasks without fear of real-world consequences. This reduced anxiety and fear allows patients to push their limits and attempt more challenging exercises, potentially accelerating recovery. In a VR environment, patients with balance disorders can practice walking on various surfaces without the risk of falling, enabling them to build confidence and improve their abilities in a secure setting [12].

Feedback Systems

XR systems can provide rich visual feedback that is impossible in traditional rehabilitation settings. This visual feedback engages the visual cortex and enhances motor learning through visual-motor integration, facilitating more effective rehabilitation. An AR system, for instance, can overlay a patient's movement trajectory with an ideal trajectory, allowing for real-time correction and improving the accuracy and efficiency of their movements [31]. Additionally, XR can incorporate auditory and haptic feedback to provide additional sensory information during rehabilitation exercises. This multimodal feedback engages multiple sensory systems, potentially enhancing motor learning and neuroplasticity. A VR system, for instance, might provide auditory cues for gait training in PD patients, combined with haptic feedback through vibrating insoles, thereby offering a more immersive and effective rehabilitation experience [32]. Supporting this, Tunik et al. (2013) reported that participants showed greater activity in the primary motor area (M1) after a stroke when provided with discordant feedback during VR training. This activation was more apparent when the feedback corresponded to the afflicted hand and recruited the contralateral M1 area. Moving the unaffected hand with virtual mirror feedback resulted in the recruitment of the ipsilateral (affected) M1 area, indicating the need for personalized visual input in VR rehabilitation [33].

Challenges and limitations

The technical challenges are hardware, software, and user interface issues that can hinder the effectiveness of XR applications. On clinical validation, extensive clinical trials are needed to validate the efficacy of XR technologies in neurological rehabilitation. For accessibility and usability, ensuring that XR technologies are accessible and user-friendly for all patients is crucial. Regarding ethical and privacy concerns, addressing data privacy and ethical considerations is essential for the responsible use of XR in healthcare.

Future directions

Regarding research and development, continued research and technological advancements are needed to optimize XR applications for neurological rehabilitation. For integration with other technologies, combining XR with AI, machine learning, and wearable devices can enhance the effectiveness of rehabilitation protocols. On policy and regulation, developing comprehensive guidelines and regulations will ensure safe and effective XR use in healthcare.

## Conclusions

This review has highlighted the potential of XR interventions in managing chronic neurological conditions by offering immersive and interactive environments that facilitate personalized rehabilitation strategies. XR technologies, including VR, AR, and MR, Improve motor function, cognitive capacities, and overall quality of life for people with illnesses such as stroke, PD, multiple sclerosis, and Alzheimer's disease. In providing engaging, contextually relevant exercises and real-time feedback, XR supports neuroplasticity and functional recovery, presenting innovative alternatives to traditional therapeutic methods.

Despite its promise, XR integration into neurological care faces several challenges, including technical issues, the need for rigorous clinical validation, and accessibility concerns. To fully realize XR's potential, future advancements must focus on refining these applications, integrating them with complementary technologies, and developing comprehensive policies to ensure ethical and effective use. As research and development progress, XR technologies are poised to revolutionize neurological rehabilitation, offering new hope and improving patient care. With thoughtful policy and regulation, XR is set to become a cornerstone of neurological therapy, transforming patient outcomes, and enhancing medical training in the years to come.
